# Deciphering the molecular basis of lipoprotein recognition and transport by LolCDE

**DOI:** 10.1038/s41392-024-02067-w

**Published:** 2024-12-27

**Authors:** Wen Qiao, Chongrong Shen, Yujiao Chen, Shenghai Chang, Xin Wang, Lili Yang, Jie Pang, Qinghua Luo, Zhibo Zhang, Yingxin Xiang, Chao Zhao, Guangwen Lu, Bi-Sen Ding, Binwu Ying, Xiaodi Tang, Haohao Dong

**Affiliations:** 1 Department of Laboratory Medicine, State Key Laboratory of Biotherapy, National Clinical Research Center for Geriatrics, West China Hospital, Sichuan University and Collaborative Innovation Center of Biotherapy, Chengdu, China; 2 Frontiers Medical Center, Tianfu Jincheng Laboratory, West China Hospital, Sichuan University, Chengdu, China; 3 Center of Cryo Electron Microscopy, Zhejiang University, Hangzhou, China

**Keywords:** Structural biology, Microbiology

## Abstract

Outer membrane (OM) lipoproteins serve vital roles in Gram-negative bacteria, contributing to their pathogenicity and drug resistance. For these lipoproteins to function, they must be transported from the inner membrane (IM), where they are assembled, to the OM by the ABC transporter LolCDE. We have previously captured structural snapshots of LolCDE in multiple states, revealing its dynamic conformational changes. However, the exact mechanism by which LolCDE recognizes and transfers lipoprotein between domains remains unclear. Here, we characterized the *E. coli* LolCDE complex bound with endogenous lipoprotein or ATP to explore the molecular features governing its substrate binding and transport functions. We found that the N-terminal unstructured linker of lipoprotein is critical for efficient binding by LolCDE; it must be sufficiently long to keep the lipoprotein’s main body outside the complex while allowing the triacyl chains to bind within the central cavity. Mutagenic assays identified key residues that mediate allosteric communication between the cytoplasmic and transmembrane domains and in the periplasmic domain to form a lipoprotein transport pathway at the LolC–LolE interface. This study provides insights into the OM lipoprotein relocation process mediated by LolCDE, with significant implications for antimicrobial drug development.

## Introduction

Gram-negative bacteria are characterized by the extra outer membrane (OM) layer, which consists of a phospholipid inner leaflet and a lipopolysaccharide outer leaflet.^1,2^ This unique asymmetry enables the OM to serve as a crucial permeable barrier, protecting bacterial cells from harmful compounds, including antibiotics.^3–5^ Lipoproteins, anchored in the OM via their N-terminally linked fatty acyl chains, are key OM components that play essential roles in OM biogenesis and maintenance of its integrity.^6–8^ OM lipoproteins facilitate the export and assembly of OM lipopolysaccharides,^9,10^ β-barrel proteins,^11,12^ and other lipoproteins.^13,14^ Additionally, they mediate the retrieval of mislocated phospholipids from the OM to the inner membrane (IM), thereby preserving the OM asymmetry.^15,16^ Some lipoproteins also keep interactions between the OM and the peptidoglycan layer to stabilize the cell envelope.^17,18^ Moreover, OM lipoproteins are critical in host-cell interaction and immune evasion,^19^ contributing to gram-negative bacteria’s high pathogenicity and antibiotic resistance, making them important targets for antimicrobial therapy.^20^ Lolamicin, a newly developed antibiotic, has been reported to target Gram-negative bacteria by specifically inhibiting the Lol lipoprotein transport system.^21^

**Fig. 1:**
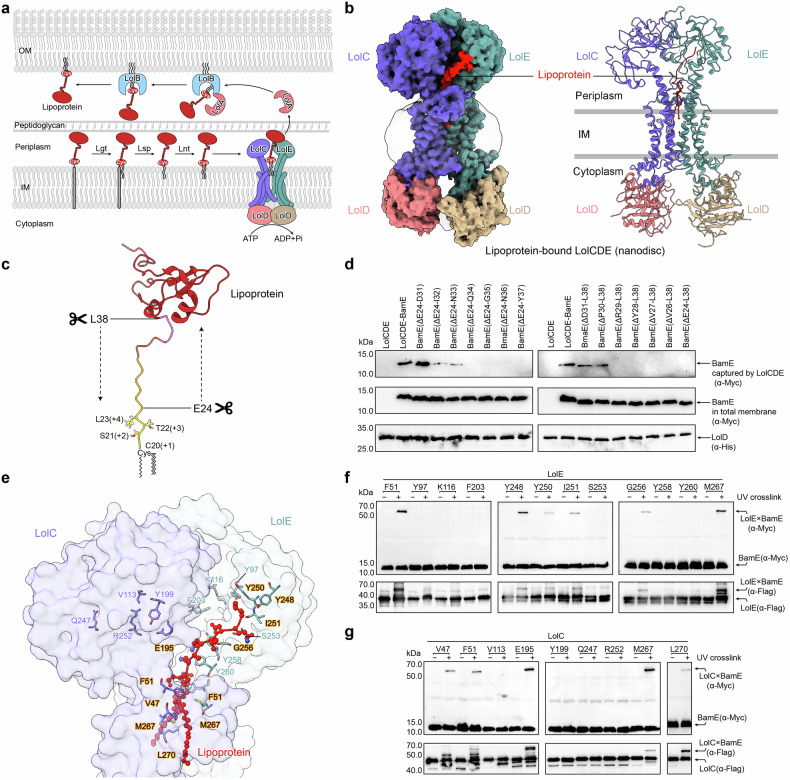
The importance of the unstructured linker of lipoprotein for efficient lipoprotein extraction by LolCDE. **a** A Schematic diagram of lipoprotein transportation by LolCDE from IM to OM. **b** Density map (left) and cartoon representation (right) of the lipoprotein-bound LolCDE structure in nanodiscs. LolC and LolE are shown in purple and green, LolD dimer is shown in pink and yellow, and lipoprotein is shown in red, respectively. **c** Cartoon representation of BamE structure (PDB code: 2KM7), indicating the truncation positions of the unstructured linker. **d** Substrate binding analysis of the truncated BamE by LolCDE. BamE was truncated from the N-terminus (left) or C-terminus (right) of the unstructured linker. **e** Residues of LolE surrounding the unstructured linker of lipoprotein and corresponding residues in LolC. **f**, **g** In vitro photo-crosslinking of lipoprotein with LolE (**f**) and LolC (**g**). Data in **d**, **f**, and **g** are representative of *n* = 3 independent experiments

OM lipoproteins are initially synthesized in the cytoplasm and then translocated to the periplasmic leaflet of the IM for maturation.^22^ In *E. coli*, the signal peptide of the lipoprotein precursor contains a ‘lipobox’ motif with a conserved cysteine residue, at which the lipoprotein precursor is cleaved and acylated with a thioether-linked diacylglycerol and an amide-linked acyl chain, resulting in a mature lipoprotein with a triacylated cysteine at the processed N-terminus^23,24^ (Fig. 1a). Depending on their roles, mature lipoproteins are either retained in the periplasmic leaflet of the IM or transported to the OM by the localization of lipoprotein (Lol) pathway, which consists of five proteins LolA-E^25^ (Fig. 1a). LolCDE forms an ATP-binding cassette (ABC) transporter in the IM, which sorts and transports lipoproteins destined for the OM by consuming energy from ATP hydrolysis.^26^ Lipoproteins containing aspartic acid (Asp) at the +2 position, adjacent to the N-terminal acylated cysteine (+1), are not transported by the Lol system (referred to as Lol avoidance signal), and therefore remain in the IM.^22,27^ LolCDE recognizes and sorts lipoproteins for their respective destinations, either in the IM or OM, based on their signal.^25,28^ Once recognized by LolCDE, OM lipoproteins are transferred to LolA, a soluble chaperon in the periplasm, through an interaction with the periplasmic domain (PD) of LolC.^29,30^ LolA then carries the lipoprotein across the periplasm and hands it off to the OM-anchored LolB, which subsequently inserts the lipoprotein into the OM^13,31^ (Fig. 1a). Photo-crosslinking assays suggest that the lipoprotein transfer from LolC to LolA and from LolA to LolB occurs in a mouth-to-mouth fashion, with their inner cavities facing each other.^32^ However, the mechanism by which LolCDE mediates lipoprotein extraction and transport to LolA through the PD of LolC remains unclear.

Our previously reported structures of various functional states of LolCDE provide insights into the allosteric mechanisms of the lipoprotein transporter.^30^ These structures demonstrate that the lipoprotein binds within the transmembrane (TM) central cavity through its N-terminal triacylcysteine and adjacent +2 and +3 position residues. Nucleotide-binding into the ATPase LolD induces the closures of the substrate central cavity and the periplasmic domains, accompanied by the disappearance of the substrate. The entire LolCDE complex undergoes significant conformational changes as it transitions from the lipoprotein-bound state to the nucleotide-bound state and finally to the unbound resting state.^30^ Despite these findings, the molecular basis of lipoprotein recognition, extraction, and translocation through each functional domain of LolCDE, particularly the periplasmic domains, remains unclear. In this study, we determined the cryogenic electron microscopic (cryo-EM) structures of endogenously captured lipoprotein- or ATP-bound LolCDE reconstituted in nanodiscs. We also identified the functional residues and characterized the molecular features of LolCDE involved in its lipoprotein recognition and export functions.

## Results

### The N-terminal unstructured linker is essential for lipoprotein binding by LolCDE

The cryo-EM structure of the lipoprotein-bound LolCDE complex reconstituted in nanodiscs was determined at a resolution of 3.5 Å, revealing a conformation highly consistent with the previously reported structure of lipoprotein-bound LolCDE in detergent (Fig. 1b and Supplementary Fig. 1). The lipoprotein is bound at the interface between the transmembrane helices (TMs) of LolC and LolE via its triacylated, unstructured N-terminal segment. Specifically, the triacyl chains and the initial (+1 to +4) residues of the lipoprotein are situated in the central cavity formed by the TM1 and TM2 of LolC and LolE, with the remaining segment extending outwards from the interface towards the periplasmic domain of LolE (Fig. 1b and Supplementary Fig. 2a). Notably, approximately half of OM lipoproteins possess a long, intrinsically disordered peptide linker at the N-terminus. This linker’s disordered nature is important for the proper relocalization of lipoproteins to the OM.^33^ To investigate whether the N-terminal linker affects the efficiency of lipoprotein binding by LolCDE, we systematically truncated the linker residues of the OM lipoprotein BamE from either end, while preserving the sorting signal residues (+1 to +4) (Fig. 1c). We then monitored the amount of BamE captured by LolCDE in vivo. Our findings indicate that at least ten residues of the N-terminal linker, including the triacylated cysteine, are necessary for efficient lipoprotein binding by LolCDE. Furthermore, the segment required for LolCDE binding appears to lack sequence specificity as truncations from either end yielded similar outcomes (Fig. 1d). Interestingly, this observation aligns with the length of the lipoprotein segment resolved in the LolCDE-lipoprotein structures, which spans from the central cavity to the periplasmic neck of TM2 in LolE (Supplementary Fig. 2b). The remaining structured body of lipoprotein has yet to be resolved in any reported LolCDE structure (Supplementary Fig. 2b), reflecting the flexibility of the unbound portion. These results indicate that LolCDE extracts lipoprotein requiring only the N-terminal triacyl chains and a small segment of the unstructured linker.

To identify LolCDE’s residues involved in lipoprotein binding, we carried out a UV-crosslinking assay on residues aligned within the path accommodating the triacyl chains and the linker in the LolCDE-lipoprotein structure (Fig. 1e). The assay revealed that residues F51 and M267 within the central cavity, G256 at the periplasmic neck of TM2, and Y248, Y250, and I251 further within the periplasmic domain of LolE effectively crosslinking with BamE (Fig. 1f). In contrast, within LolC, only the central cavity residues V47, F51, and M267, along with a periplasmic loop residue E195 near the TM2 neck of LolE, exhibited crosslinking with BamE (Fig. 1g). Notably, no crosslinking was observed at the periplasmic neck residues of TM2 in LolC. These results are consistent with prior binding analysis, which implicated LolE, but not LolC, in the interaction with lipoprotein.^34^ These results suggest that the triacyl chains of the lipoprotein interact with cavity residues in both LolE and LolC, whereas the lipoprotein linker is predominantly recognized by LolE via the periplasmic residues of TM2. This finding is consistent with the common feature observed in all reported lipoprotein-bound LolCDE structures, where the N-terminal linker of the lipoprotein is tilted towards LolE^30,35,36^ (Supplementary Fig. 2b).

The structural surface of the complex reveals highly hydrophobic patches surrounding the central cavity formed by TM1 and TM2, as well as the interface created by the gate loops extended between TM3 and TM4 (Supplementary Fig. 2c). Each of the triacyl chains (designated R1, R2, and R3) binds specifically within these hydrophobic pockets. The thioester-linked acyl chain, R1, is situated in the interface enclosed by the gate loop of LolC, while the amide-linked acyl chain, R3, and the other thioester-linked acyl chain, R2, are accommodated in a larger pocket enclosed by the gate loop (formerly known as the door bar) of LolE (Supplementary Fig. 2d). These specific interactions of the lipid chains and peptide linker to the complex indicate that the LolCDE extracts lipoprotein in a fixed orientation, allowing each structural element of the lipoprotein molecule to be precisely recognized by different domains of LolCDE.

### LolC T302 and LolE T307 are involved in allosteric communications between the NBDs and TMDs

**Fig. 2:**
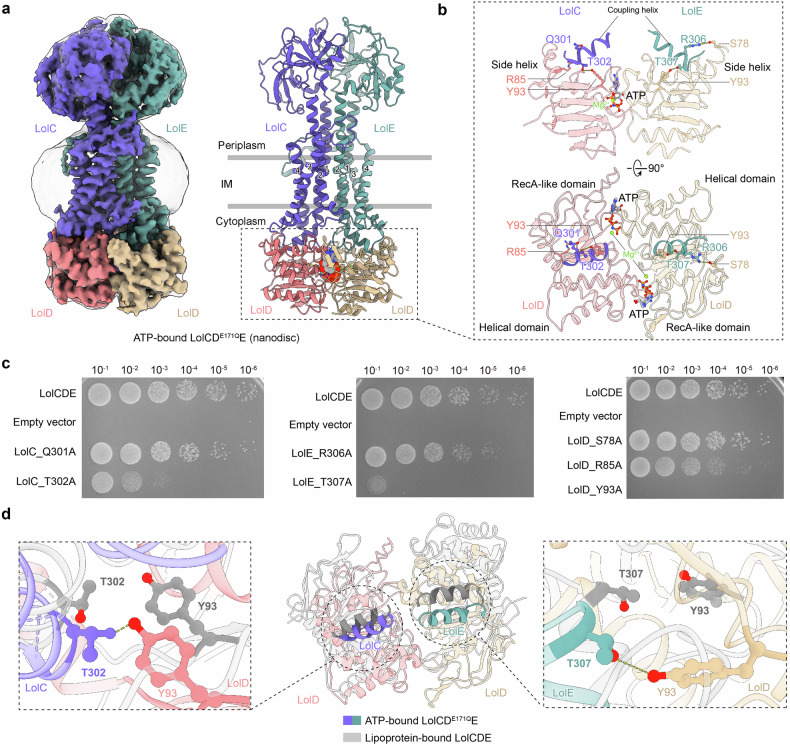
Identification of the allosteric communicating residues of NBDs and TMDs of the LolCDE complex. **a** Cryo-EM density map (left) and cartoon representation (right) of LolCD^E171Q^E structures in nanodiscs with ATP binding at 3.2 Å resolution. LolC and LolE are shown in purple and green, LolD dimer is shown in pink and yellow, and ATP is shown in gray, respectively. **b** Side and top views of the NBDs showing interactions with the coupling helices of LolC and LolE. The bound endogenic ATP molecules and magnesium ions are indicated. **c** Cell viability of the LolCDE mutants of the coupling helix interacting residues. Data are representative of *n* = 3 independent experiments. **d** Structural superimposition of the coupling helices of LolC (left) and LolE (right) and the NBDs of the lipoprotein-bound and ATP-bound LolCDE structures, showing distinct interactions at the coupling helices between the two structures

Our previous finding revealed that the ATPase catalytic mutant LolCD^E171Q^E reduced lipoprotein binding, whereas the ATP-binding mutant LolCD^K48A^E abolished lipoprotein transport in vitro^30^, implicating that lipoprotein transportation is triggered by ATP binding rather than hydrolysis. To investigate the molecular basis of ATP-bound LolCDE, we determined the structure of the mutant complex LolCD^E171Q^E. The structure of *E. coli* LolCD^E171Q^E reconstituted in nanodiscs was determined to 3.2 Å using cryo-EM (Fig. 2a and Supplementary Fig. 3). Despite the absence of added nucleotides, additional cryo-EM densities were observed in the catalytic pockets of LolD, which were identified as ATP molecules and magnesium ions (Fig. 2a and Supplementary Fig. 3). The overall conformation of the LolCD^E171Q^E structure closely resembles the structures of LolCDE bound to ATP analogs, AMP-PNP and ADP-vanadate, with root-mean-square deviations (RMSD) of 1.2 Å over 405 aligned residues and 1.0 Å over 393 aligned residues, respectively^30,35^ (Supplementary Fig. 4a).

The nucleotide-binding domains (NBDs) of LolCD^E171Q^E adopt a closed conformation, tightly sandwiching the endogenously captured ATP molecules between the RecA-like subdomain and the α-helical subdomain of opposing LolD subunits (Fig. 2b). Comparing the ATP-bound LolCD^E171Q^E structure with the lipoprotein-bound structure, LolD dimerization induced remarkable conformational changes in the transmembrane domains (TMDs) and periplasmic domains (PDs) (Supplementary Fig. 4b). The NBDs interact with the TMDs through the coupling helices of LolC and LolE, which fit into the grooves between the RecA-like and α-helical subdomains of LolD (Fig. 2b). In the ATP-bound LolCD^E171Q^E structure, a pair of polar residues, Q301 and T302, from the coupling helix of LolC interact with R85 on the side helix (S78-Q87) and Y93 on the Q-loop (I92-A104) of one LolD subunit, while R306 and T307 from the coupling helix of LolE interact with S78 on the side helix and Y93 on the Q-loop of the other LolD, respectively (Fig. 2b). To assess the importance of these polar residues to LolCDE’s function, we substituted them with alanine and conducted cellular viability assays (Fig. 2c). Since lipoprotein transport by LolCDE is an essential process in bacteria, disrupting the function of LolCDE leads to cellular lethality. The mutagenic assays were carried out using our previously reported *lolCDE* null strain HD200313, in which the expression of the *lolCDE* is controlled by the araBAD promotor.^30^ The results showed that the single mutant T302A in LolC, T307A in LolE, or Y93A in LolD, which are involved in the interactions between the coupling helix of LolC or LolE and the Q-loop of LolD, impaired bacterial viability (Fig. 2c and Supplementary Fig. 4c). These mutations did not affect the expression of the LolCDE complex (Supplementary Fig. 4d). Interestingly, these interactions were absent in the lipoprotein-bound structure, where the NBDs were in an undimerized conformation (Fig. 2d). This finding suggests that these interactions are crucial for coupling the conformational changes between the NBDs and TMDs in response to ATP binding.

### LolC W249 and LolE W254 couple the conformational changes at the TMD-PD interface

**Fig. 3:**
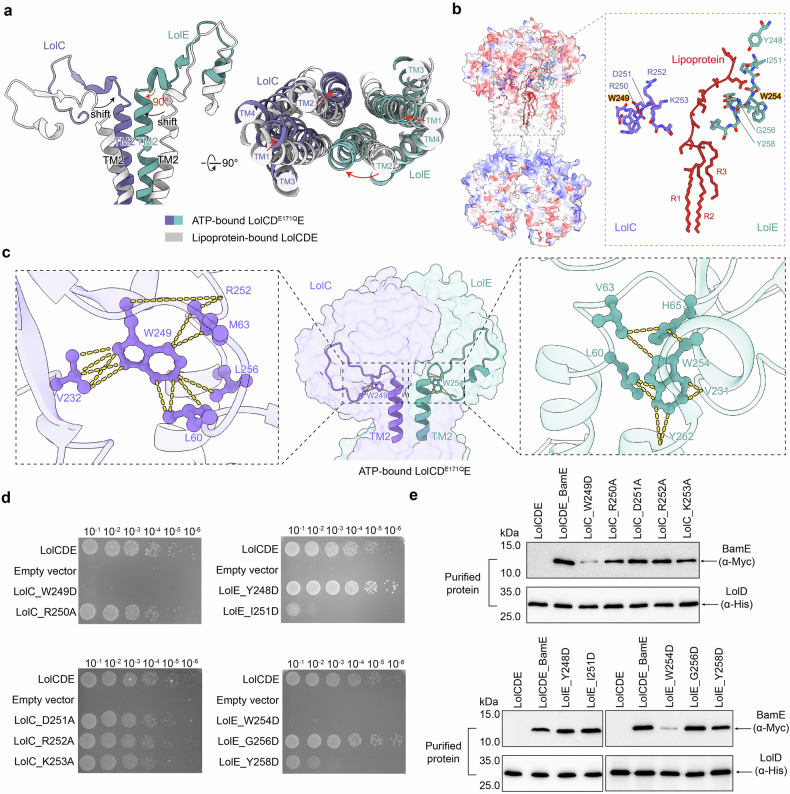
Conformational change of the TM2 periplasmic neck of LolC and LolE in lipoprotein- and ATP-bound structures. **a** Structural superimposition of the lipoprotein-bound and ATP-bound LolCDE structures reveals conformational changes between TM helices of LolC and LolE. **b** Residues of the TM2 periplasmic necks of LolC and LolE are indicated. **c** A pair of tryptophan at the bend of the TM2 periplasmic neck of LolC and LolE, showing multiple contacts with surrounding residues in LolC (left) and LolE (right). **d** Cell viability of the mutants shown in (**b**). **e** Detections of the lipoprotein BamE binding to the mutant complexes shown in (**d**) by western blotting. Data in **d**, **e** are representative of *n* = 3 independent experiments

When comparing the TMDs of the ATP-bound and the lipoprotein-bound structures, the most striking conformational changes are observed in the long transmembrane helices TM1 and TM2 (Fig. 3a). In the ATP-bound structure, TM1 and TM2 of both LolC and LolE are more parallel, creating a closed interface as opposed to the V-shaped cavity observed in the lipoprotein-bound structure (Fig. 3a). This closed interface between LolE and LolC is incompatible with lipoprotein binding, as the TM2 helix of LolE in the ATP-bound LolCD^E171Q^E structure would clash with the triacyl chains present in the lipoprotein-bound structure (Supplementary Fig. 5a). These observations suggest that the ATP-bound LolCD^E171Q^E structure represents a post-transport state, where the bound lipoprotein is expelled from the central cavity as a result of the cavity closure induced by ATP binding. In this structure, TM2 of LolE undergoes an inward and upward movement, resulting in an approximately 90-degree bend at the periplasmic neck, transitioning from a relatively straight conformation in the lipoprotein-bound structure to a bent conformation in the ATP-bound state (Fig. 3a). Conversely, TM2 in LolC already exhibits a bent conformation in the lipoprotein-bound structure, with the state transition causing only a shift (Fig. 3a). Intriguingly, the periplasmic residues along TM2 of LolE and LolC display distinctly different chemical properties: while LolC residues are predominantly charged (W^249^RDRKGE^255^), those in LolE are primarily hydrophobic (W^254^IGTYGY^260^) (Fig. 3b). Notably, both LolC and LolE contain a conserved tryptophan residue, LolC^W249^and LolE^W254^, symmetrically positioned at the bending point of the TM2 neck (Fig. 3b), establishing extensive intermolecular contacts with surrounding residues from TM1, TM2, and the PDs (Fig. 3c). In particular, the aromatic side chain of LolC^W249^ interacts with L60 of TM1, R252 and L256 of TM2, and V232 of the periplasmic loop inLolC (Fig. 3c). Similarly, the aromatic side chain of LolE^W254^ makes contacts with L60, V63, and H65 of TM1, Y262 of TM2 and V231 of the periplasmic loop in LolE (Fig. 3c). Similar interactions involving LolC^W249^ and LolE^W254^ are also observed in the lipoprotein-bound structure (Supplementary Fig. 5b); however, these contacts are absent in the apo LolCDE structure (PDB code: 7ARI).

To evaluate the importance of these periplasmic residues for LolCDE’s function, we modified their chemical properties through site-directed mutagenesis and assessed their effects on rescuing the LolCDE null strain (Fig. 3d). Among the mutations examined, the mutations W254D in LolE and W249D in LolC completely abolished bacterial viability (Fig. 3d and Supplementary Fig. 5c–e). Additionally, the purified mutant protein complexes of LolCDE^W254D^ and LolC^W249D^E exhibited diminished lipoprotein binding (Fig. 3e). These suggest that LolCDE^W254^ and LolC^W249^E are critical for the lipoprotein extraction function of LolCDE. Previous studies have shown that lipoprotein extraction by LolCDE occurs following ATP hydrolysis,^30,35,36^ which resets the LolCDE complex back to its relaxed apo conformation and disrupts the surrounding membrane to facilitate lipoprotein entry into the cavity. Notably, LolE^W254^ and LolC^W249^ do not directly interact with the bound lipoprotein, suggesting that their stabilized conformation between the TMDs and PDs is essential for efficient lipoprotein extraction.

We also investigated the periplasmic residues, LolE^I251^ or LolE^Y258^, which are located along TM2 and associated with the unstructured linker. The mutants LolE^I251D^ or LolE^Y258D^ exhibited reduced bacterial viability, but the purified mutant proteins showed normal lipoprotein binding (Fig. 3d, e and supplementary Fig. 5d, e). The milder effects suggest that these residues are important, but not essential for the function of LolCDE. In contrast, mutations in the TM2 residues on LolC showed no noticeable effect either in vivo or in vitro (Fig. 3d, e and Supplementary Fig. 5c, e).

### Lipoprotein exits from the central core of the PDs for exportation

**Fig. 4:**
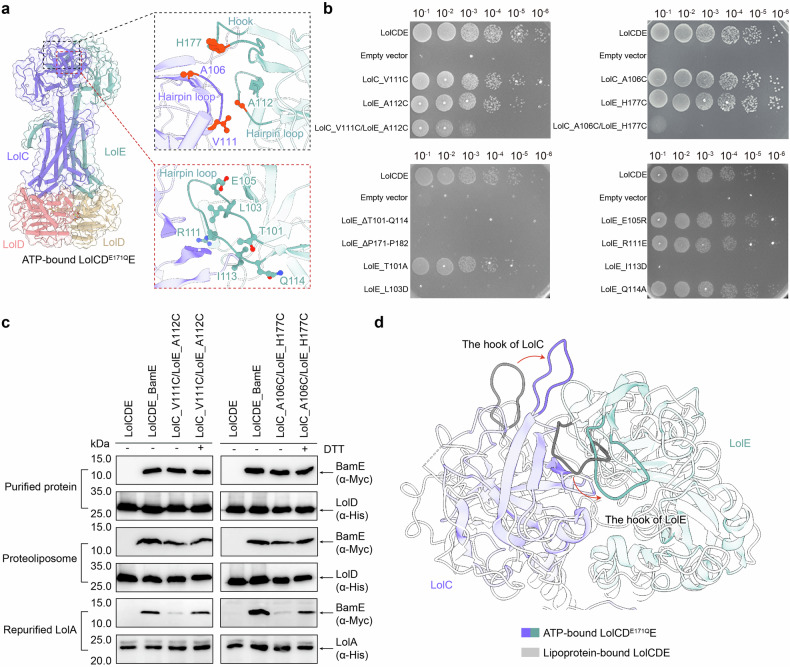
Analysis of lipoprotein export path between the periplasmic domains of LolCDE. **a** The hydrophobic cavity formed by the periplasmic cores of LolC and LolE in the ATP-bound structure of LolCDE. The hairpin loop of LolCE and the hook of LolE are highlighted with the indication of the residues mutated for the disulfide-bonded crosslink. **b** Cell viability of the mutants shown in (**a**). **c** In vitro lipoprotein transport assay of the disulfide-bonded mutants LolC^V111C^DE^A112C^ and LolC^A106C^DE^H177C^ to LolA. Data in **b**, **c** are representative of *n* = 3 independent experiments. **d** Conformational changes of the hooks of LolC and LolE in the lipoprotein-bound and ATP-bound structures

The periplasmic domains (PDs) of the ATP-bound LolCD^E171Q^E structure exhibit prominent conformational changes compared to the lipoprotein-bound structure (Fig. 4a and Supplementary Fig. 6a). Each PD comprises two subdomains, Sabre and Porter,^30^ which together form a central core (Supplementary Fig. 6b). The shifts of TM2 elevate the base of the PDs, causing the periplasmic cores of LolC and LolE to enclose a cavity with the interior hydrophobic β-sheets facing each other (Supplementary Fig. 6b). To investigate whether these β-sheets constitute the path for lipoprotein export, we disrupted their continuity by introducing disulfide bonds between two opposing Sabre β-strands in LolC and LolE at the center. We then performed an in vivo viability assay and an in vitro lipoprotein transport assay to evaluate the effects. The in vitro transport assays were conducted according to a previously established protocol, using purified LolCDE reconstituted in phospholipid liposomes mixed with LolA, ATP, and magnesium ions to facilitate lipoprotein transport in vitro.^30^ Structural and biochemical studies indicate that the purified wild-type LolCDE complex naturally binds a lipoprotein in the substrate cavity, with lipoprotein delivery to LolA occurring in the presence of ATP and MgCl_2_.^30,37,38^ Cysteine mutations were introduced at residues LolC^V111C^ and LolE^A112C^, which are in close proximity (less than 4 Å), on the β strands of LolC and LolE, respectively (Fig. 4a). Electrophoresis confirmed that the purified LolC^V111C^DE^A112C^ mutant forms a disulfide-bonded complex, which could be reduced in the presence of DTT (Supplementary Fig. 6c, d). The in vivo analysis showed that the LolC^V111C^DE^A112C^ mutant significantly diminished bacterial viability compared to the unaffected single cysteine mutations at LolC^V111C^ or LolE^A112C^ (Fig. 4b and Supplementary Fig. 6e). In vitro transport studies demonstrated that the crosslinked LolC^V111C^DE^A112C^ complex reconstituted in phospholipid liposomes completely abolished lipoprotein transport to LolA when compared to the wild-type (Fig. 4c and Supplementary Fig. 6c, d). These results suggest that lipoprotein must traverse the central hydrophobic space enclosed by the β-sheets of the Sabre subdomains during its export to LolA.

Furthermore, disrupting the integrity of this hydrophobic path by removing one side of the β strands proved detrimental (Fig. 4a, b). Deletion of the hairpin T101–Q114, which contains the interacting portion of the β strands from the Sabre subdomain of LolE, resulted in complete lethality without affecting the expression of the protein complex (Fig. 4a, b and Supplementary Fig. 6e, f). Notably, substituting the hydrophobic residue L103 or I113 with the charged residue aspartic acid within the Sabre β-strand of LolE was sufficient to cause lethality, whereas substituting the nearby polar residue T101 or Q114 by alanine had no effect (Fig. 4b and Supplementary Fig. 6e, f). Similarly, reversing the charges of E105 or R111 in the Sabre β-sheet of LolE had no effect (Fig. 4b and Supplementary Fig. 6e, f). These findings confirm that the hydrophobic pathway formed by the β-sheets of PDs is critical for LolCDE’s function, likely by accommodating the triacyl chains of the transported lipoprotein.

The PD of LolC associated with the periplasmic protein LolA via the hairpin hook structure for lipoprotein delivery.^29^ In the ATP-bound structure, the hook of LolC lifts and shifts towards the interface between the PDs, positioning itself to be accessible by both LolA and the bound lipoprotein (Fig. 4d). For lipoprotein transport to occur, the lipoprotein bound to the PD of LolE needs to ultimately reach the PD of LolC. However, the hook of LolE appears to obstruct the interface between the PDs of LolC and LolE, as it is situated close to an opposite periplasmic loop of LolC (Fig. 4d). This observation raises the question of whether an unimpeded interface between the PDs is essential for lipoprotein export. To test it, we crosslinked the proximate loops by introducing a disulfide bond through cysteine mutations at the LolE hook residue H177C and the LolC loop residue A106C (Fig. 4a). The purified LolC^A106C^DE^H177C^ protein forms a disulfide-bonded complex that is reducible by DTT (Supplementary Fig. 6d, g). The LolC^A106C^DE^H177C^ mutant was lethal in vivo and inhibited lipoprotein transport to LolA in vitro (Fig. 4b, c and Supplementary Fig. 6d, e, and g). These results suggest that the delivery of lipoprotein to LolA necessitates passage through the interface between the PDs. The flat conformation of the LolE hook observed in the ATP-bound structure may play a role in preventing lipoprotein backflow. Additionally, deletion of the LolE hook segment P171–P182 also resulted in lethality (Fig. 4b and Supplementary Fig. 6e). However, the expression of the LolCDE^∆P171-P182^ mutant protein was markedly reduced (Supplementary Fig. 6f), indicating that the hook of LolE is crucial for structural stability of the complex.

### Identification of lipoprotein binding residues in the periplasmic core of LolCDE

**Fig. 5:**
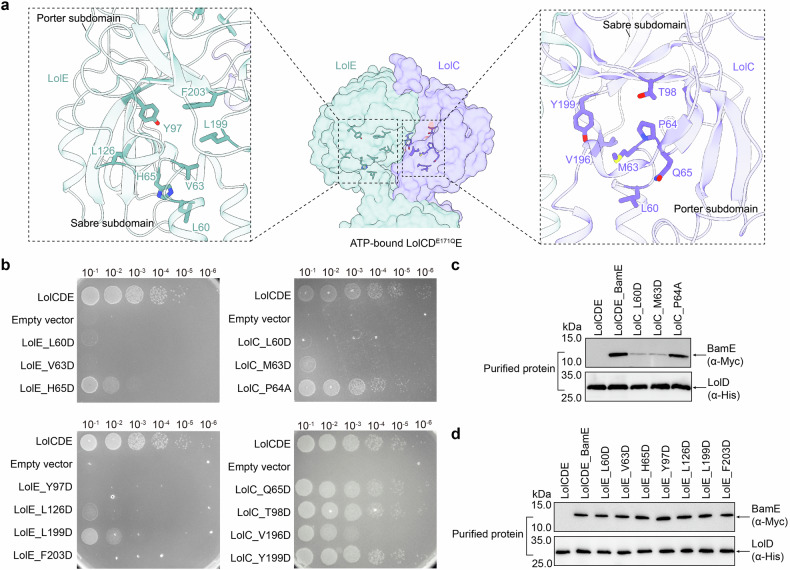
Functional analysis of the periplasmic central core of LolCDE. **a** Cartoon representation of the periplasmic domains of LolE (left) and LolC (right) showing the hydrophobic residues at the periplasmic central cores. **b** Cell viability of the mutants shown in (**a**). **c**, **d** Lipoprotein binding analysis in the mutant complexes shown in (**b**) by western blot. Data in **b**, **c** are representative of *n* = 3 independent experiments

The conformational changes in the periplasmic necks of transmembrane helices caused the PDs to move closer together, with the PD of LolE exhibiting the most pronounced alternations (Supplementary Fig. 4b and Supplementary Fig. 6a). In the ATP-bound structure, the helmet-shaped PDs of LolC and LolE come together to create a periplasmic cavity. Both the PDs of LolC and LolE contain a hydrophobic core, which connects with the hydrophobic cavity of the TMDs (Supplementary Figs. 2d and 7a). To determine whether the residues in the hydrophobic core are essential for the functionality of LolCDE (Fig. 5a), we performed single-site mutagenesis and assessed cellular viabilities. Substituting the residues L60, V63, and H65 in the periplasmic neck of TM1 in LolE with aspartic acid (L60D, V63D, and H65D) led to lethality or severe viability defects (Fig. 5a, b and Supplementary Fig. 7b). Additionally, mutations L199D and F203D from the Sabre subdomain, as well as Y97D and L126D in the region between the Sabre and Porter subdomains of LolE, also resulted in bacterial death or viability defects (Fig. 5b and Supplementary Fig. 7b). Western blot analysis confirmed that the expression levels of the mutant proteins were not affected (Supplementary Fig. 7c), suggesting that the inhibitory effects were due to functional defects rather than protein expression defects. These results indicate that the internal residues of the periplasmic core of LolE are crucial for the function of LolCDE. In contrast, the mutants in the periplasmic residues of LolC had milder effects. Only the L60D and M63D mutations in TM1 of LolC caused severe viability defects (Fig. 5b and Supplementary Fig. 7b, d), whereas the V196D and Y199D mutations within the Sabre subdomain, and T98D in the region between the Sabre and Porter subdomains, had minimal impact on bacterial viability (Fig. 5b and Supplementary Fig. 7b, d). Interestingly, the L60D and M63D mutants in LolC also showed abolished lipoprotein binding (Fig. 5c), whereas lethal mutants in LolE did not affect lipoprotein binding (Fig. 5d). L60 and M63 are located on the TM1 periplasmic neck of LolC and interact with W249 on TM2 (Fig. 3c). Disrupting the hydrophobicity of L60, M63, and W249 consistently led to lethality and loss of lipoprotein binding (Figs. 3c and 5c), suggesting that the periplasmic conformation of LolC stabilized by these hydrophobic interactions is essential for lipoprotein extraction. In contrast, mutations at L60, V63, and H65, which interact with W254 of LolE at the periplasmic neck, also caused lethality but maintained relatively normal lipoprotein binding levels. This suggests that these hydrophobic residues of LolE have functions beyond lipoprotein binding, likely contributing to the stabilization of the conformational changes of the PD required for lipoprotein transport.

## Discussion

Lipoproteins are key components of the outer membrane in Gram-negative bacteria. Correct sorting and relocation of newly synthesized lipoproteins by LolCDE is fundamental for bacterial viability.^28,39^ Given its essential role, LolCDE presents a promising target for therapeutical intervention against bacterial infections.^21^ In our previous work, we reported several cryo-EM structures of LolCDE in different working conformations, providing structural insights into the allosteric mechanism of this lipoprotein transporter.^30^ In the present study, we further explored the molecular communications within the structural domains of LolCDE and identified key residues crucial for its function in lipoprotein transport. By disabling the ATPase activity of LolD, we successfully captured an endogenous ATP-bound LolCDE complex. Although the conformation of the ATP-bound structure aligns with previously characterized complexes bound to ATP analogs, our structure confirms the physiologically relevant state of a nucleotide-bound LolCDE complex, assisting in studying the molecular mechanism of LolCDE.

In the cytoplasmic region, we identified a pair of threonine residues, LolC T302 and LolE T307, symmetrically positioned on the coupling helix of LolC and LolE, respectively, which facilitate the allosteric communications between the ATP-bound NBDs and TMDs (Fig. 2b). Structural analysis reveals that LolE undergoes more significant conformational changes than LolC upon ATP binding, consistent with the reported asymmetry in their action.^35^ This observation led us to question why the dimerization of the homodimer LolD induces asymmetric allostery in the TMDs of LolC and LolE. Interestingly, we found that two additional residues S412 and R18 located on the C- and the N-terminal elbow helices of LolE interact with the opposite LolD unit at S144 and R142 on the signature loops at the front and back of the complex, respectively (Supplementary Fig. 7e). Such interactions are not seen in LolC. These additional couplings between LolE and LolD may contribute to the asymmetric conformational changes between LolE and LolC. However, mutagenesis of these interacting residues did not confirm their functional importance, likely because some of these hydrogen bonds originate from the main chains of the residues, making residue substitution ineffective in disrupting their interactions (Supplementary Fig. 7e).

Previous work by our group and others has elucidated the molecular details involved in the binding of triacylcysteine moiety of lipoprotein within the central cavity of LolCDE, highlighting the strong hydrophobic extractive force that LolCDE exerts on the lipid portion of lipoprotein.^30,35,36^ In our current study, we further explore the significance of the N-terminal unstructured linker in lipoprotein extraction. Through UV crosslinking and BamE truncation experiments, we confirmed that the binding of the unstructured linker is exclusively mediated by LolE. The N-terminal linker must be sufficiently long to interact with the PD of LolE, facilitating efficient binding via the TM2 helix (Fig. 1c, d). The neck of TM2, leading to the PD of LolE, is rich with hydrophobic residues, providing a broad region (W254-Y262) for hydrophobic interactions with the sequence-unspecific linker (Fig. 3b). Specifically, a pair of tryptophan W254 and W249 on the TM2 periplasmic neck of LolE and LolC, respectively, engage extensively with surrounding residues (Fig. 3c). Substitutions of W254 of LolE and W249 of LolC resulted in severe cellular lethality and a significant decrease in lipoprotein binding. These findings implicate that the interaction interfaces formed by LolE W254 and LolC W249 are crucial for lipoprotein extraction. In bacteria such as *E. coli*, over 90 different OM lipoproteins are produced.^22,40^ Despite varying in the C-terminal functional groups, they share a common N-terminal triacylated cysteine. The extraction of lipoproteins through this conserved triacylcysteine and the unstructured linker allows diverse lipoproteins to be transported by the single LolCDE pathway. Interestingly, the minimal linker length required for efficient lipoprotein extraction coincides with the distance from the triacyl binding cavity to the periplasmic edge of LolE TM2. This observation suggests that the presence of the linker ensures the exclusion of the bulky functional group of lipoproteins outside of the LolCDE complex while enabling the terminal triacyl chains to reach the PDs for effective levering out of the cavity.

From the lipoprotein-bound to the ATP-bound state, the PDs of LolC and LolE undergo significant conformational changes that are conducive to lipoprotein export. Firstly, the hook of LolC protrudes, making it accessible to the periplasmic acceptor LolA. Secondly, the long β-strands in the Sabre subdomain of LolC shift to align better with the β strands flanking the hook, forming a relatively smooth β-sheet that guides the lipoprotein toward the hook. Thirdly, the PD of LolE in the ATP-bound state reorients its buried β-sheets to face those of LolC, creating a new hydrophobic cavity that may temporarily accommodate the triacyl chains during the transfer of lipoprotein to LolA. Tethering the hydrophobic cavity via a disulfide bond between the facing β sheets at the central core of LolC and LolE inhibited lipoprotein transport, suggesting that the central hydrophobic core in PDs forms a path for lipoprotein export. Interestingly, in both the lipoprotein-bound and ATP-bound structures, the hook of LolE remains in a closed conformation, covering the interface between the two PDs. This configuration does not seem to impede the translocation of the lipoprotein from the LolE side to the LolC–LolA side. When the hook of LolE was cross-linked to the opposite loop of LolC, lipoprotein transport was diminished in vitro, confirming that access between the PDs is required for effective lipoprotein transportation. It remains unclear whether the lipoprotein squeezes through the narrow gap beneath the hook of LolE or whether the hook of LolE momentarily opens and then returns to its closed conformation after the lipoprotein is delivered. Notably, the deletion of the LolE hook compromised the stability of the complex, underscoring its role in maintaining structural integrity.

**Fig. 6:**
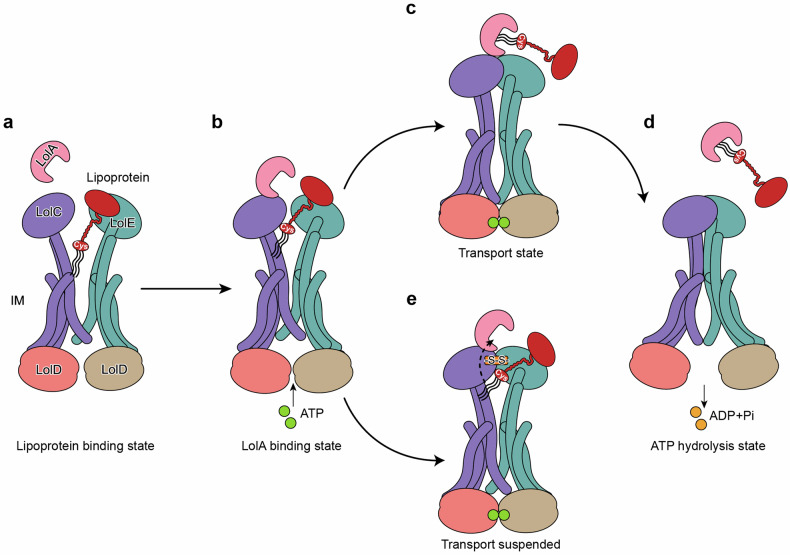
Mechanism of LolCDE-mediated lipoprotein transport. **a** The lipoprotein binding state: a lipoprotein is bound at the central cavity of LolCDE via the N-terminal triacyl chains and the unstructured linker. **b** The LolA-binding state: the chaperone protein LolA binds to the complex via the periplasmic domain of LolC. **c** The transport state: ATP binding induces dimerization of the nucleotide-binding domains, which closes the transmembrane cavity to expel the bound lipoprotein. **d** The ATP hydrolysis state: ATP hydrolysis resets LolCDE back to the relaxed apo conformation, which may facilitate lipoprotein extraction for the next cycle. **e** Crosslinking of the periplasmic domains of LolCDE by disulfide bond blocks the lipoprotein transport

In summary, our study elucidates the molecular mechanisms at play within the NBDs and PDs of LolCDE, particularly in the recognition and translocation of lipoproteins (Fig. 6). These findings complement previously reported insights regarding the binding of lipoprotein triacyl chains within the TMDs of LolCDE.

## Methods

### Expression and purification of LolCDE, LolCD^E171Q^E and LolA_21-203_

The plasmid pTRC99a-*lolCDE* from *E. coli* K-12 was constructed as previously described,^30^ with an octa-histidine (8× His) tag at the C terminus of LolD. To generate of LolCD^E171Q^E mutation (where the E171 residue of LolD is mutated to glutamine), site-directed mutagenesis^41^ was performed using the LolCDE plasmid as the template in a polymerase chain reaction (PCR).

The plasmids encoding LolCDE and LolCD^E171Q^E were individually transformed into *E. coli C43* (DE3) cells (Novagen) for protein expression. The cells were grown at 37 °C in LB medium containing 100 μg ml^−1^ ampicillin until the OD_600_ reached 0.8. Protein expression was then induced with 0.2 mM Isopropyl β-D-thiogalactoside (IPTG) at 20 °C for 12 h.

Cells were harvested and resuspended in lysis buffer A (50 mM Tris-HCl, pH 7.8, 300 mM NaCl, 10% (*v*/*v*) glycerol) containing 0.1 mM phenylmethylsulfonyl fluoride (PMSF). The cells were lysed, and cell membranes were isolated by ultracentrifugation at 100,000 × *g* for 1 h at 4 °C. The resulting membrane pellets were solubilized in buffer A containing 1% (*w*/*v*) n-dodecyl-β-d-maltopyranoside (DDM; Anatrace) at room temperature for 1 h, followed by another round of ultracentrifugation to removed insoluble material. The supernatant containing the solubilized protein was applied to HisTrap HP column (5 ml, GE HealthCare) pre-equilibrated with buffer A containing 0.05% (*w*/*v*) lauryl maltose neopentyl glycol (LMNG; Anatrace). The resins were washed with buffer A containing 40 mM imidazole and 0.05% LMNG. The bound protein was then eluted using buffer A supplemented with 300 mM imidazole and 0.05% LMNG. Further purification was carried out using size-exclusion chromatography (SEC) on a Superdex 200 Increase 10/300 column (GE Healthcare) in buffer B (50 mM Tris-HCl, pH 7.8, 150 mM NaCl) supplemented with 0.05% LMNG. The LolCDE protein fractions were pooled and concentrated to 4 mg ml^−^^1^ using a 100-kDa cutoff Amicon Ultra-15 (Merck-Millipore).

The plasmid pET28a-LolA_21–203_ containing an N-terminally octa-histidine (8× His) tag was used to express the periplasmic LolA_21-203_ protein, as described previously.^30^ In brief, *E. coli* BL21 (DE3) cells were used to express LolA_21-203_, and the cells were lysed. The lysate was subjected to ultracentrifugation at 17,000 × *g* for 1 h at 4 °C to remove unbroken cell debris. The clarified lysate containing LolA was purified by a Ni-NTA column. The resin was washed with Buffer A containing 300 mM imidazole, and excessive imidazole was removed via a desalting column (Hi-Prep 26/10, GE Healthcare). The purified protein was concentrated to 10 mg ml^−1^ using a 10-kDa cutoff Amicon Ultra-15 (Merck-Millipore), flash frozen, and stored at −80 °C until use.

### Reconstitution of LolCDE and LolCD^E171Q^E into lipid nanodiscs

*E. coli* polar lipids (Avanti Polar Lipids), solubilized in chloroform, were dried under nitrogen gas and re-dissolved in lipid dilution buffer (20 mM Tris-HCl, pH 7.8, 150 mM NaCl, 100 mM sodium cholate). LMNG-solubilized LolCDE (or LolCD^E171Q^E), MSP1D1, and the lipid mixture were combined at a molar ratio of 1:2:200, with the concentration of cholate maintained at approximately 40–50 mM, and incubated at 4 °C for 1 h. To remove the detergent, the mixture was treated with SM2 Bio-Beads (Bio-Rad) for 3 h at 4 °C. The sample was then purified using a Superdex 200 Increase 10/300 column (GE Healthcare) equilibrated with buffer B (50 mM Tris-HCl pH 7.8, 150 mM NaCl). The purified protein in nanodiscs was collected for cryo-EM sample preparation.

### Reconstitution of the LolCDE-BamE complexes in proteoliposomes

The pTRC99a*-lolCDE-BamE* construct, which includes a Myc-tag at the C terminus of BamE, was generated as previously described in ref.^30^ LolCDE-BamE and its relevant mutations were expressed and purified following the same methods as those used for LolCDE, and utilized for detecting the bound BamE via western blot, as well as for proteoliposome reconstitution.

*E. coli* polar lipid extract (Avanti Polar Lipids) was first solubilized in chloroform and then dried under nitrogen gas to form a lipid film. The lipid films were resuspended in buffer B (50 mM Tris-HCl, pH 7.8, 150 mM NaCl) at a concentration of 10 mg ml^−1^, sonicated, and then passed through a polycarbonate membrane with a pore size of 0.4 μm to create liposomes. To destabilize the liposomes, 1.6 mmol LMNG per mg of lipid was added, followed by incubation with purified wild-type or mutant LolCDE-BamE at a molar ratio of 20:1 for 1 h on ice. Detergent was removed and protein was incorporated into the liposomes by diluting the mixture 150 fold with buffer B. Proteoliposomes were then pelleted by ultracentrifugation at 300,000 × *g* for 30 min at 4 °C and resuspended at a concentration of 1 mg ml^−1^ in buffer B. The wild-type or mutant LolCDE–BamE proteoliposomes were prepared for transport assays and western blot analysis.

### In vitro photo-crosslinking assay

To capture the interactions between LolCDE components and substrate lipoprotein, *E.coli* BL21(DE3) strains were transformed with the plasmids of pSup-BpaRS-6TRN,^42^ pTRC99a-*lolCDE-BamE*, with amber (TAG) codons introduced in either LolC or LolE. These strains were grown at 37 °C in LB containing 100 μg ml^-1^ ampicillin and 25 μg ml^-1^ chloramphenicol. When OD_600_ reached 0.5, LolCDE-BamE protein was induced by adding 20 μM IPTG, 0.1% (*w*/*v*) l-arabinose, and 0.5 mM *p*-benzoyl-l-phenylalanine (pBPA, Bachem). Cells were harvested after 2 h of overexpression at 37 °C. The LolCDE-BamE complexes were solubilized and purified by nickel-affinity chromatography, following the same procedure as for LolCDE. The purified proteins were divided into two halves: one half was irradiated with UV light (λ = 365 nm) on ice for 20 min, while the other half remained untreated.

For western blot analysis, the samples were separated by sodium dodecyl-sulfate polyacrylamide gel electrophoresis (SDS-PAGE) and transferred to PVDF membranes (Bio-Rad). Crosslinked adducts were detected using a mouse anti-Flag monoclonal antibody (1:1000 dilution; F3165, Sigma) and a mouse anti-Myc monoclonal antibody (1:1000 dilution; A5963, Sigma).

### In vitro transportation assay

To monitor lipoprotein transport, a one-cycle in vitro lipoprotein transport assay was performed as previously described.^30^ Wild-type or mutant LolCDE-BamE proteoliposomes were mixed with purified LolA_21-203_ at a molar ratio of 1:2 in buffer C (50 mM Tris-HCl, pH 7.8, 150 mM NaCl, 1 mM ATP and 2 mM MgCl_2_). The reactions were carried out at 4 °C for 20 min. To stop the reaction, 7.5 volumes of ice-cold buffer B were added, and LolCDE-BamE or its mutant reconstituted proteoliposomes, along with LolA_21-203_, were separately by centrifugation at 300,000*×*g for 30 min at 4 °C. The supernatant containing LolA_21-203_ was further purified using a Ni-NTA column to detect captured BamE by western blot. The collected proteoliposomes were resuspended in buffer B for western blot analysis.

### Cell viability assays

The *E. coli* knock-in strain HD200313, with LolCDE expression controlled by the araC-araBAD promoter system, was previously described.^30^ For mutagenesis, the plasmid pTRC99a-*lolC*_*(Flag)*_*DE*, pTRC99a-*lolCD*_*(His)*_*E* or pTRC99a-*lolCDE*_*(Myc)*_ were used as templates in PCR to introduce single mutations, allowing protein expression to be detected by Western blotting. All mutations were generated following the site-directed mutagenesis protocol as previously described.^41^ *E. coli* HD200313 cells were transformed with the pTRC99a-*lolCDE* plasmid or its mutants, and the transformed cells were grown on LB supplemented with 100 μg ml^−1^ ampicillin and 0.2% l-arabinose at 37 °C for 12 h. Cells were collected and washed twice with LB to remove l-arabinose. The pellets were resuspended in fresh LB and adjusted to an OD_600_ to 0.5, and the cells were diluted 10-fold to create a concentration gradient from 10^−1^ to 10^−6^. Then 5 μl of the diluted cells were spotted onto LB agar plates containing 100 µg ml^−1^ ampicillin, with or without 0.05% l-arabinose, and grown overnight at 37 °C. All assays were performed in triplicate.

Wild-type or mutated LolCDE with leaky expression were purified and detected by western blot using anti-Flag (1:1000 dilution; F3165, Sigma), anti-Myc monoclonal antibody (1:1000 dilution; A5963, Sigma), or anti-His (1:1000 dilution; SAB2702218, Sigma) with secondary rabbit anti-mouse immunoglobulin-G (IgG) antibody. Protein bands were visualized by chemiluminescence in a photo imager (Cytiva).

### Electron microscopy sample preparation and data collection

A 3 μl of freshly purified LolCDE or LolCD^E171Q^E in nanodiscs, at a concentration of approximately 5 mg ml^−1^, was applied to glow-discharged Quantifoil holey-carbon grids (R1.2/1.3, 300 mesh, copper). The grids were blotted for 3.5 s at 95% humidity using a Vitrobot Mark IV (Thermo Fisher Scientific) before being plunge-frozen in liquid ethane cooled by liquid nitrogen. Cryo-EM images were acquired on a Titan Krios electron microscope (Thermo Fisher Scientific) equipped with a K2 Summit electron counting direct detection camera (Gatan). All images were collected in counting mode at a nominal magnification of 29,000×, corresponding to a calibrated physical pixel size of 1.014 Å. The defocus range was set between −1.0 μm to −2.8 μm. Each image was captured over an exposure time of 8 s, dose fractionated into 40 frames, with a dose rate of about 7.8 counts per second per physical pixel.

### Image processing

Beam-induced motion was corrected using the program MotionCor2,^43^ which generated average dose-weighted micrographs. The contrast transfer function (CTF) parameters of these average micrographs were estimated with Gctf.^44^ Data processing was carried out using RELION^45^ and cryoSPARC.^46^ For LolCDE in nanodiscs, 1,398,358 particles were automatically selected, and consistent particle classes were further refined through 2D and 3D classifications, resulting in 192,234 particles being used for 3D refinement. The final map, with a resolution of 3.5 Å, was obtained after post-processing with a B factor of −108 Å^2^. Detailed data-processing steps are shown in Supplementary Fig. 1.

For LolCD^E171Q^E in nanodiscs, 1,281,615 particles were automatically selected and processed through 2D and 3D classification, with 268,413 particles used for final 3D refinement. The resulting map, with a resolution of 3.2 Å was generated after post-processing with a B factor of −60 Å^2^. Further details are presented in Supplementary Fig. 3.

### Model building and refinement

The atomic model of lipoprotein-bound LolCDE in a nanodisc, with a resolution of 3.5 Å was built using the structure of lipoprotein-bound LolCDE in detergent (PDB code: 7ARH) as a docking model.^30^ This structure closely resembles the lipoprotein-bound LolCDE in detergent, and clear density for the lipoprotein density is visible. Iterative rounds of real-space refinement were carried out in PHENIX,^47^ and the overall structure was further improved by manual adjustments in Coot.^48^

Similarly, the atomic model of ATP-bound LolCD^E171Q^E in a nanodisc, with a resolution of 3.2 Å was built using the structure of AMP-PNP bound LolCDE (PDB code: 7ARK) as a starting model. Two additional density regions corresponding to ATP molecules were identified at the LolD catalytic sites, which are endogenous and bound to the NBDs. These ATP molecules were manually fitted into the densities map using Coot, followed by iterative real-space refinement rounds in PHENIX. Notably, in the ATP-bound LolCD^E171Q^E map, no lipoprotein densities were observed, suggesting that lipoprotein is not bound to LolCDE in this conformation.

All structures underwent further manual adjustments and automatic refinement using PHENIX with phenix.real_space_refine. The refinement statistics are listed in Supplementary Table 1. Structural figures were prepared using Chimera and ChimeraX.^49^

## Supplementary information

Supplementary figures

PDB validation report for ATP-bound LolCD(E171Q)E

PDB validation report for Lipoprotein-bound LolCDE

## Data Availability

The data that support the findings of this study are openly available in PDB at https://rcsb.org, EMDB at https://www.ebi.ac.uk/emdb/, reference number 9GRC (lipoprotein-bound LolCDE) and 9GVK (ATP-bound LolCD^E171Q^E), EMD-51520 and EMD-51637.
