# Supplementary material for: Deciphering the molecular basis of lipoprotein recognition and transport by LolCDE
Source: Signal Transduct Target Ther. 2024 Dec 27;9:354. doi: 10.1038/s41392-024-02067-w (PMC11671585; doi:10.1038/s41392-024-02067-w)
Supplement: Supplementary file 1 — Supplementary figures [file 41392_2024_2067_MOESM1_ESM.docx]

**Supplementary Materials for**

**Deciphering the molecular basis of lipoprotein recognition and transport by LolCDE**

Wen Qiao^1,^ ^2, #^, Chongrong Shen^1, #^, Yujiao Chen^1, #^, Shenghai Chang^2^, Xin Wang^1^, Lili Yang^1^, Jie Pang^1^, Qinghua Luo^1^, Zhibo Zhang^1^, Yingxin Xiang^1^, Chao Zhao^1^, Guangwen Lu^1^, Bisen Ding^1^, Binwu Ying^1,^*, Xiaodi Tang^1,^*, Haohao Dong^1,2,^*

^#^These authors contributed equally to this work.

*Correspondence: yingbinwu@scu.edu.cn (B.Y.), Xiaodi Tang (tangxiaodi@scu.edu.cn) or Haohao Dong (haohaodong@scu.edu.cn).

**This PDF file includes:**

Figures. S1 to S7

Tables S1

**
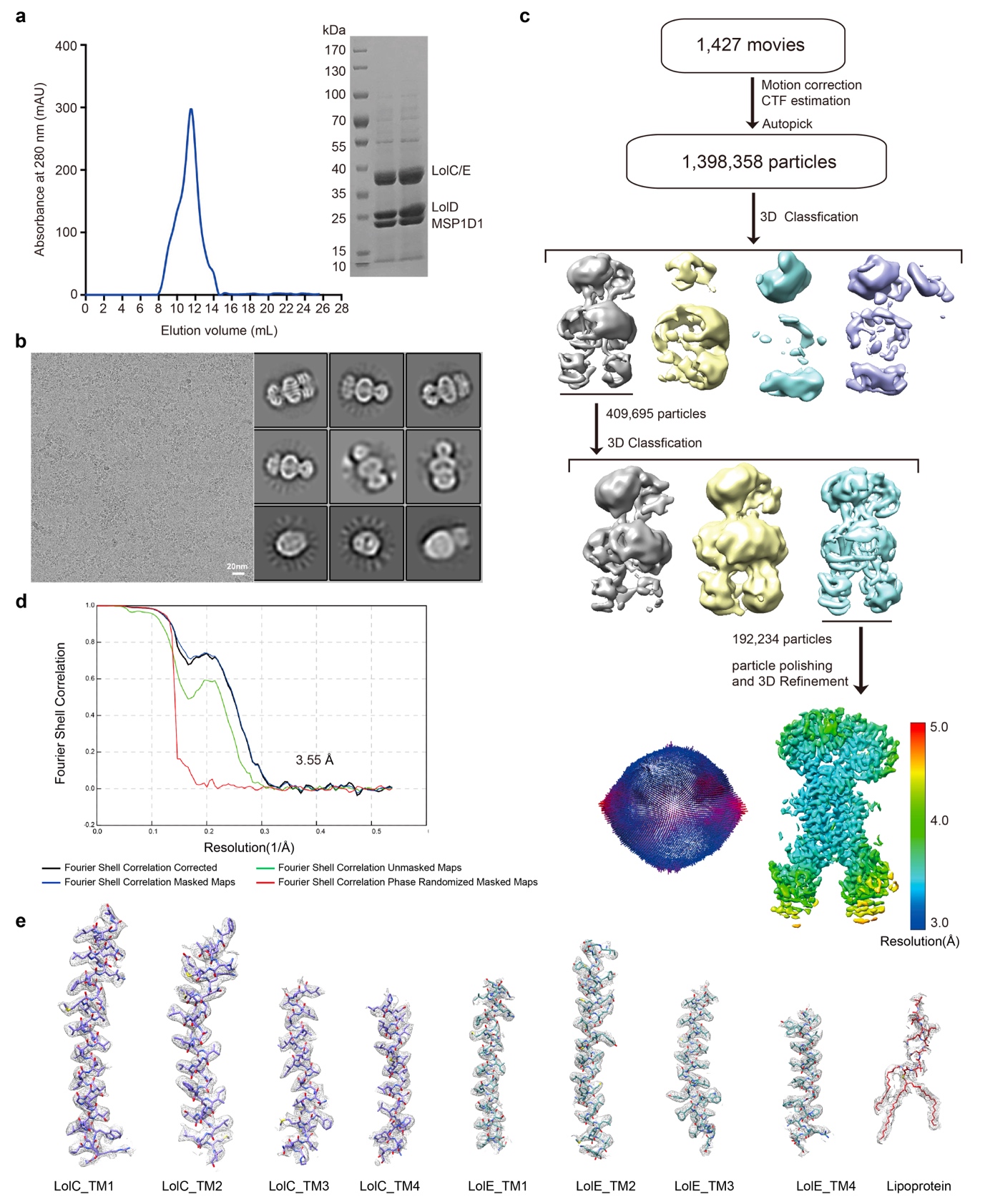
 Figure. S1 Flowchart for cryo-EM single-particle data processing of lipoprotein-bound lolCDE in nanodiscs. a** The size-exclusion chromatogram and SDS-PAGE analysis of purified LolCDE reconstituted in nanodiscs. **b** Cryo-EM microscope and selected two-dimensional images of lipoprotein-bound LolCDE. **c** Three-dimensional classification and refinement of cryo-EM particle images, and the final resolutions of the density map for lipoprotein-bound LolCDE. **d** The gold-standard FSC curves of the final Cryo-EM maps. **e** Cryo-EM maps with the atom model for TM1-TM4 of LolC and LolE and the lipoprotein.


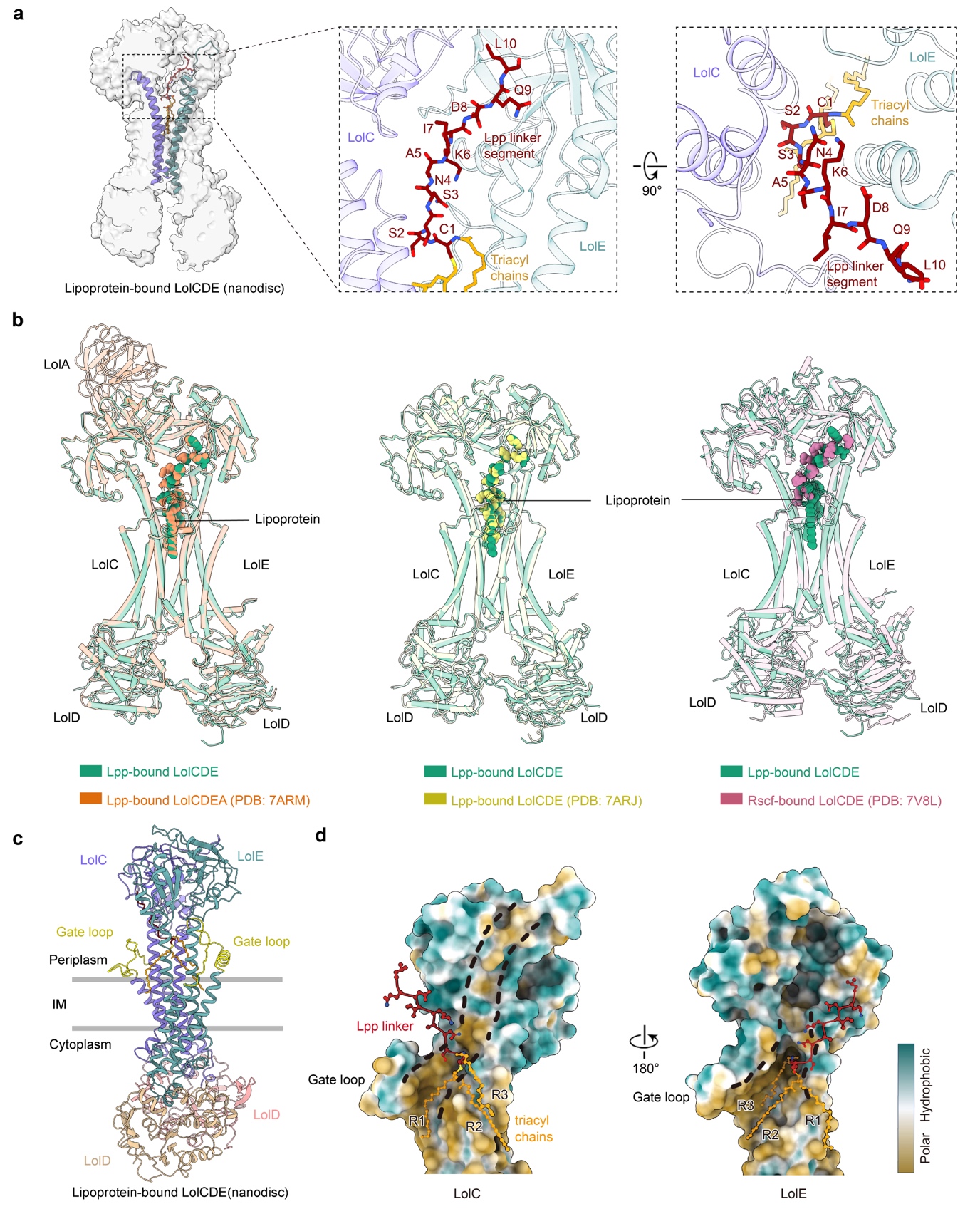


**Figure S2 Cartoon structure of LolCDE binding lipoprotein.** **a** The details of lipoprotein bound in LolCDE. **b** Structural superimposition of our lipoprotein-bound LolCDE structure with lpp bound LolCDEA (PDB code: 7ARM), lpp and AMP-PNP bound LolCDE (PDB code: 7ARJ) and rscf bound LolCDE (PDB code: 7V8L). **c** The conformations of lipoproteins bound to LolC and LolE, respectively. The gate loops of LolCE are highlighted in yellow.


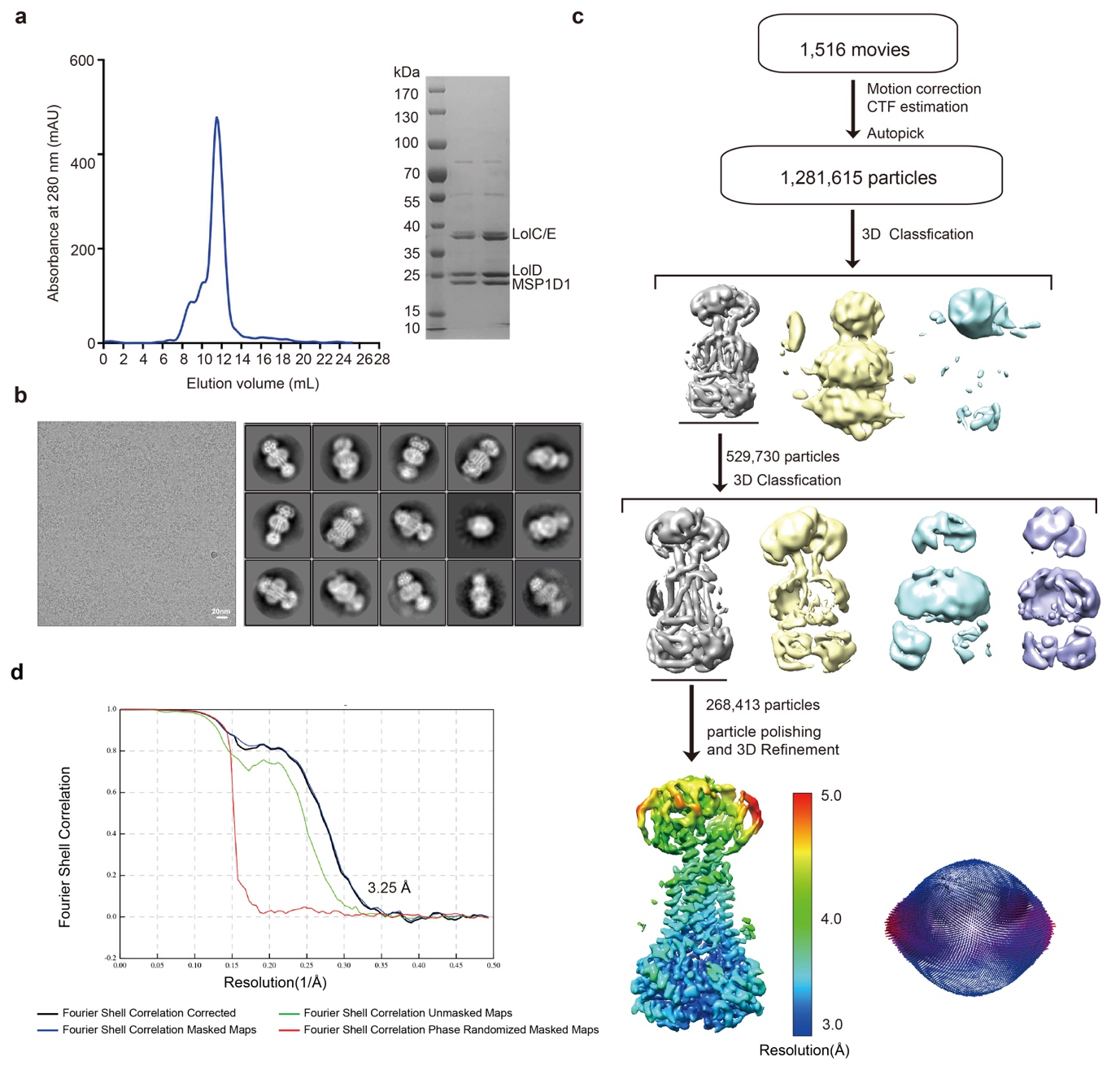


**Figure S3 Flowchart for cryo-EM single-particle data processing of ATP-bound lolCDE in nanodiscs. a** The size-exclusion chromatogram and SDS-PAGE analysis of purified LolCD^E171Q^E reconstituted in nanodiscs. **b** Cryo-EM microscope and selected two-dimensional images of ATP-bound LolCDE. **c** Three-dimensional classification and refinement of cryo-EM particle images, and the final resolutions of the density map for ATP-bound LolCDE. **d** The gold-standard FSC curves of the final Cryo-EM maps.


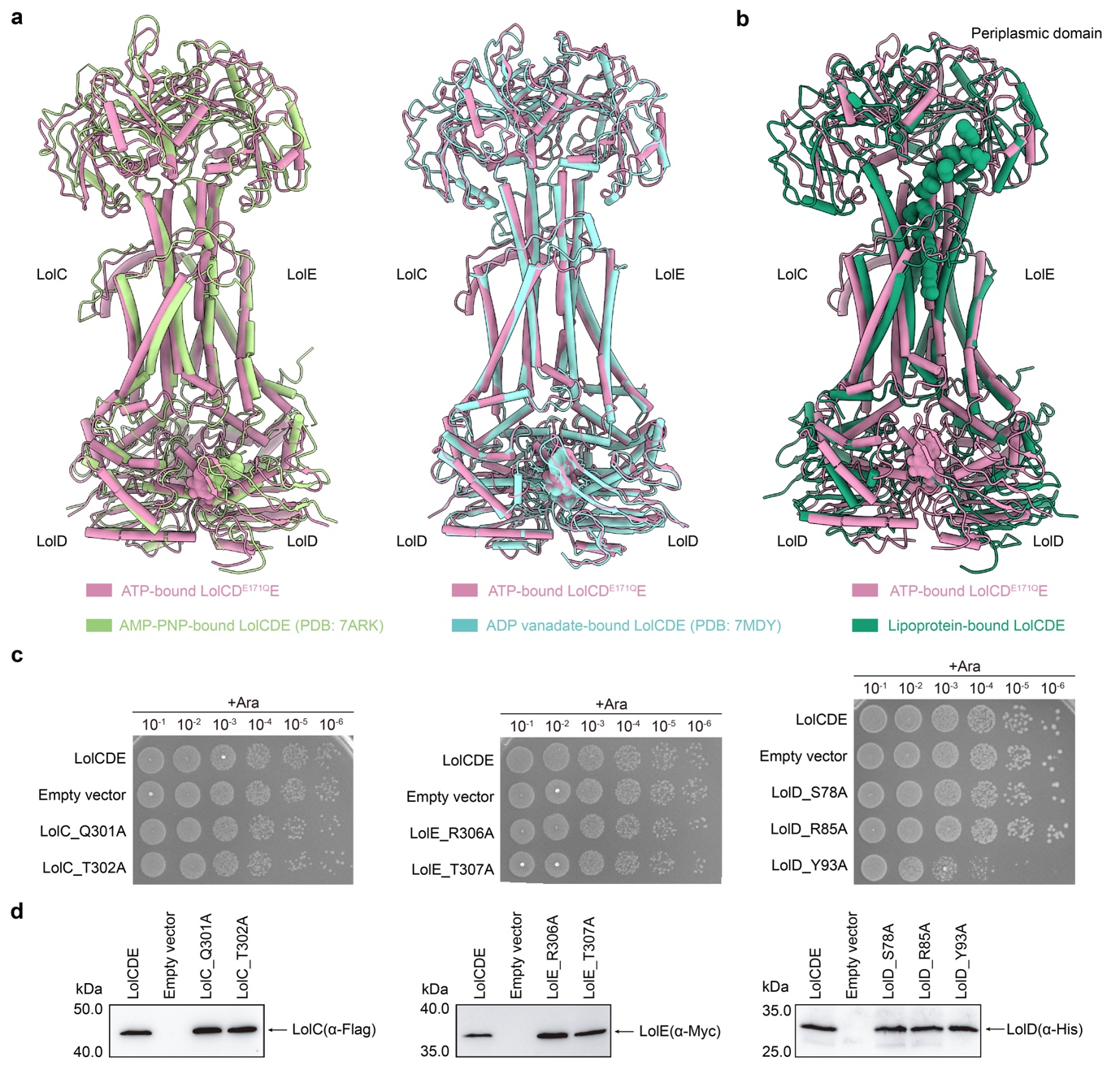


**Figure S4 Conformational changes of LolCDE upon nucleotide-bound. a** Structural superimposition of our ATP-bound LolCDE structure with the AMP-PNP bound LolCDE (PDB code: 7ARK) and the ADP bound LolCDE (PDB code: 7MDY). **b** Comparison of cartoon structures of ATP-bound and lipoprotein-bound LolCDE. **c** Cell viability of the LolCDE mutants rescued by arabinose-induced protein expression. **d** Western blot detection of wild-type and mutant LolCDE shown in **c**. Data in c-d are representative of n=3 independent experiments.


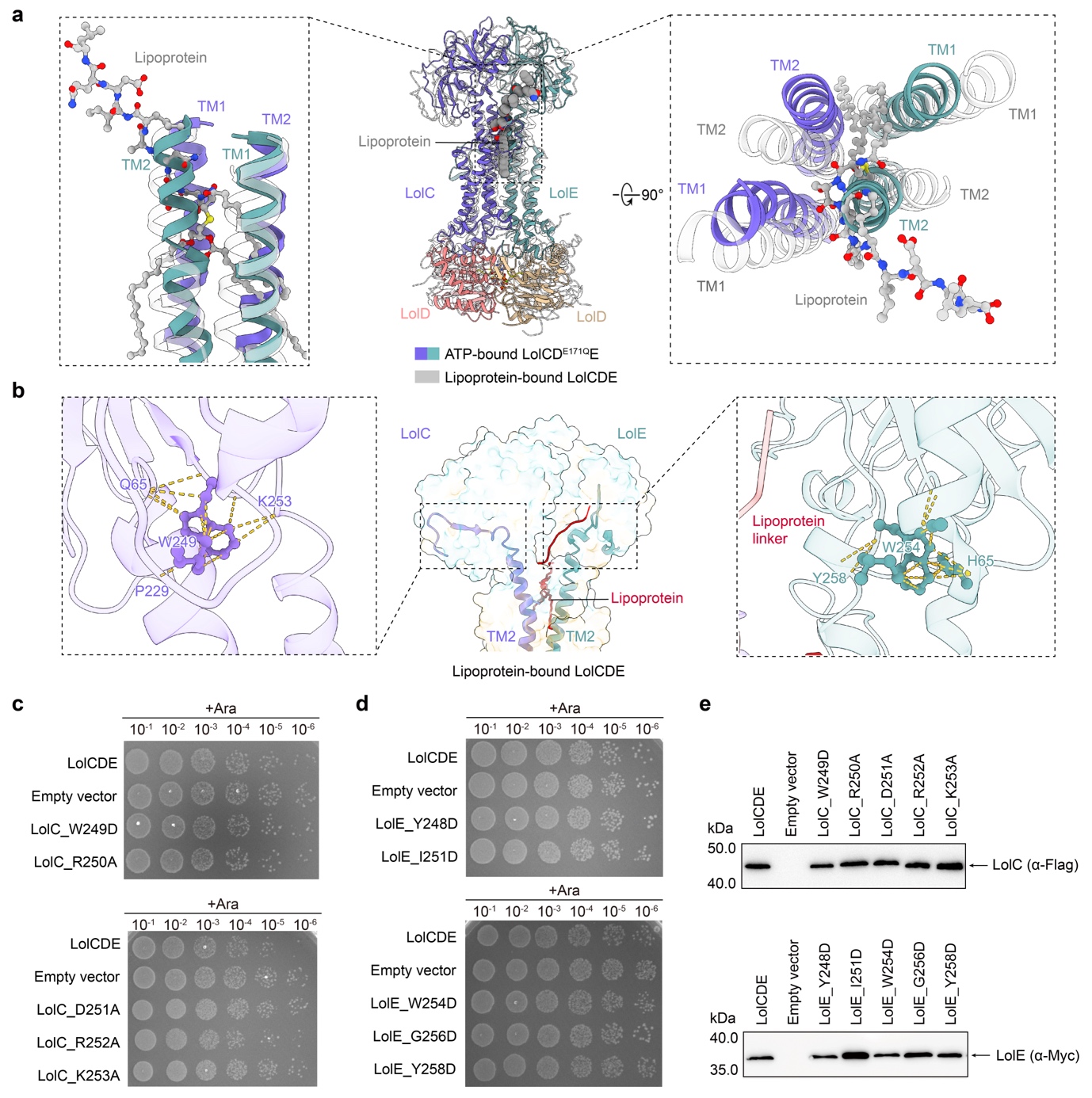


**Figure S5 Conformational changes of** **TM2 periplasmic neck of LolC and LolE. a** The lipoprotein is clash with the TM2 of LolE in ATP-bound LolCD^E171Q^E structure. **b** W249 of LolC (right) and W254 of LolE (left) on the TM2 neck forms multiple interactions with the surrounding residues. **c** Cell viability of the mutants of the TM2 periplasmic neck residues of LolE (Y248A, I251D, W254D, G256D, Y258D) rescued by arabinose-induced protein exression. **d** Cell viability of the mutants of TM2 periplasmic neck residues of LolC (W249D, R250A, D251A, R252A, K253A). **e** Western blot detection of of wild-type and mutant LolCDE as shown in (c,d). Data in c-e are representative of n=3 independent experiments.


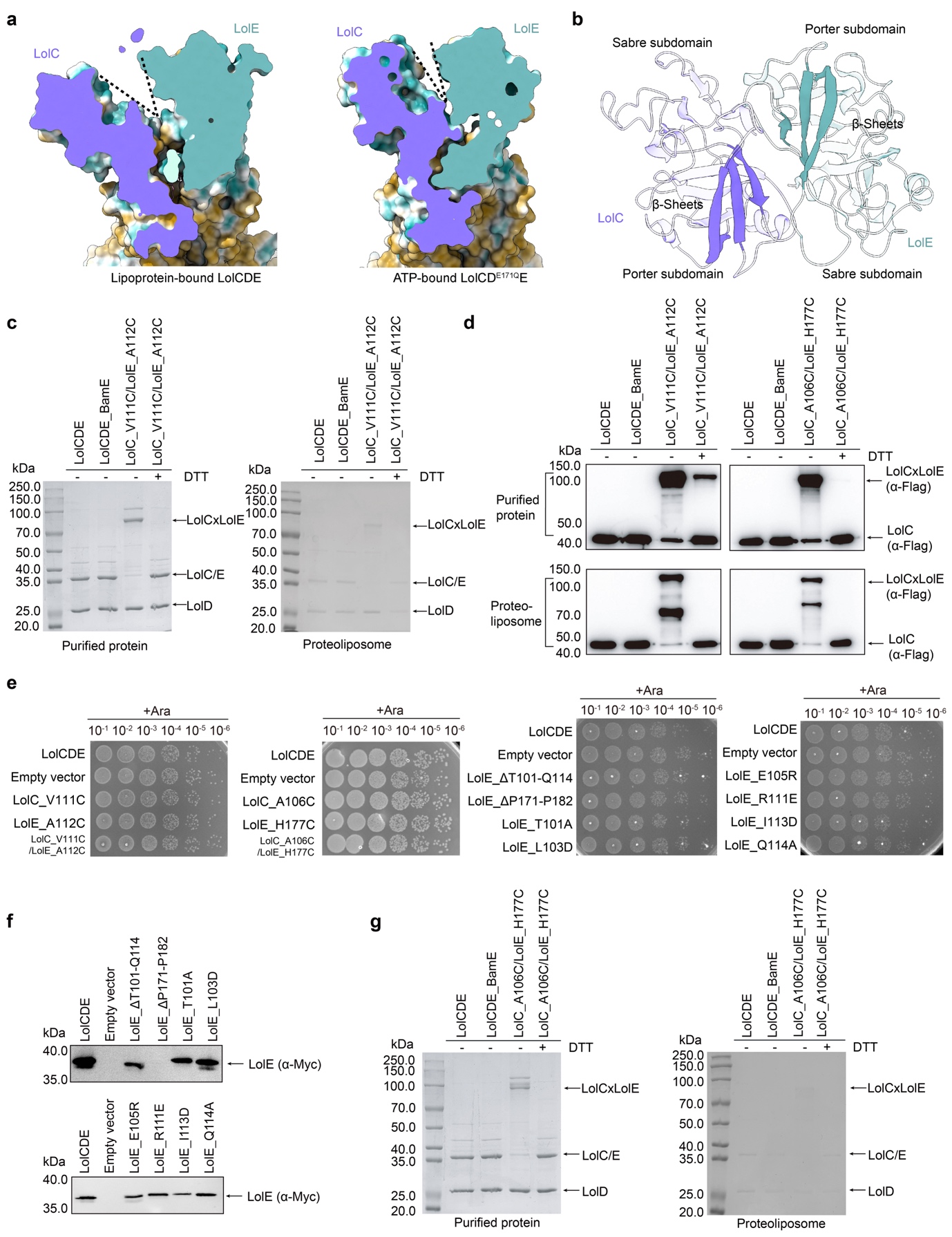


**Figure S6** **Pathway of lipoprotein transport through the PD of LolCDE.** **a** The surface representation of PD cavity of lipoprotein-bound (left) and ATP-bound (right) LolCDE. **b** Conformation of the interior hydrophobic β-sheets in PDs of ATP-bound LolCDE. **c** SDS-PAGE analysis of purified proteins of wild-type LolCDE with or without BamE and PD residues mutants LolC^V111C^DE, LolCDE^A112C^, LolC^V111C^DE^A112C^ in both detergent and liposomes. **d** Western blot detection of purified proteins of wild-type with or without BamE and cysteine mutations in or not in the presence of DTT. **e** Cell viability of cysteine mutations and truncations of LolE, and mutants of LolE periplasmic hairpin residues rescued by arabinose-induced protein expression. **f** Western blot detection of protein expression including wild-type and mutants shown in (e). **g** SDS-PAGE analysis of purified proteins of wild-type LolCDE with or without BamE and mutants in periplasmic hooks (LolC^A106C^DE, LolCDE^H177C^, LolC^A106C^DE^H177C^) in both detergent and liposomes. Data in c-g are representative of n=3 independent experiments.


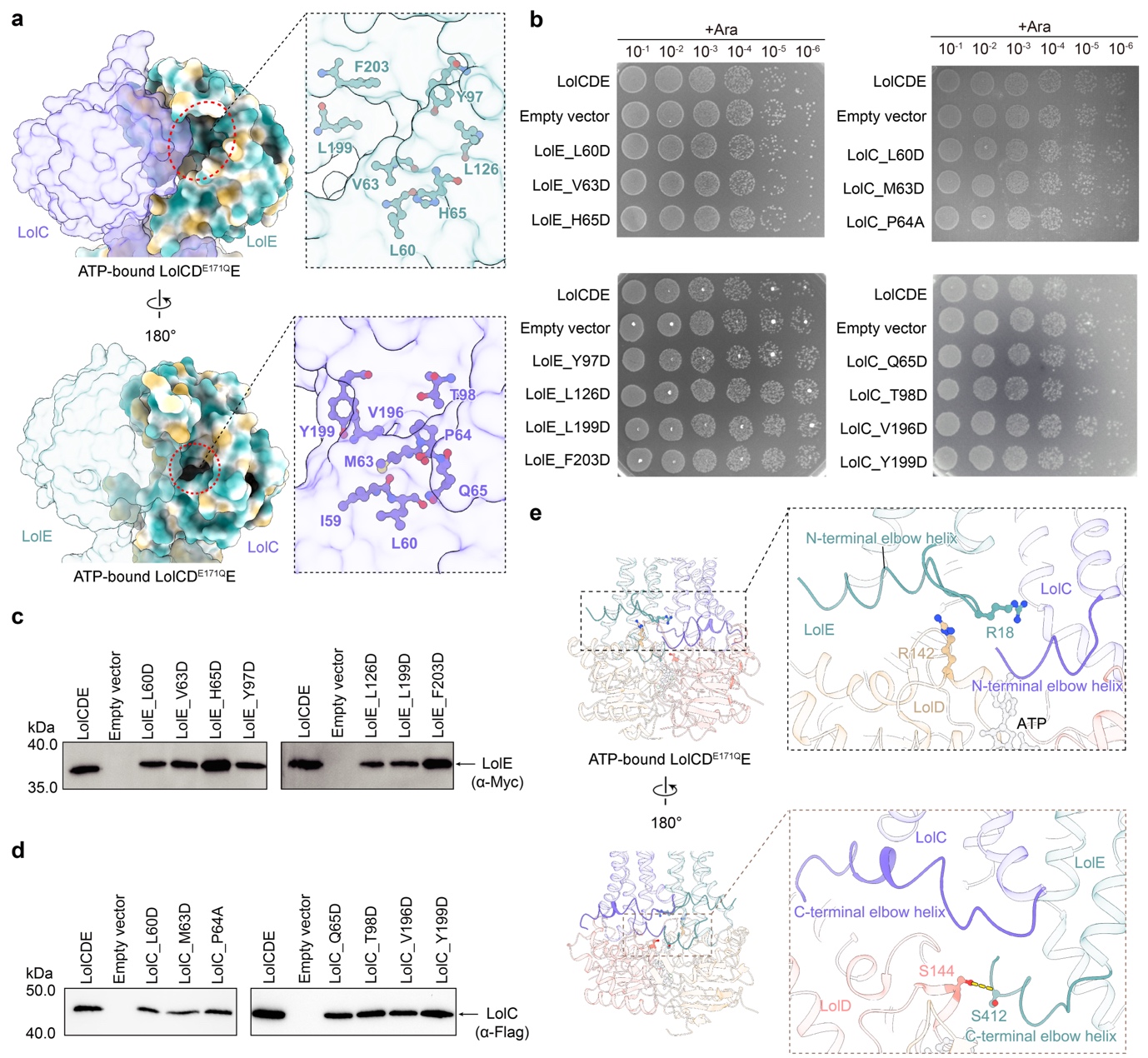


**Figure S7 | Functional analysis of the periplasmic central cores of LolE and LolC. a** The periplasmic central cores of LolE (left) and LolC (right) shown in hydrophobic surface. **b** Cell viability of the mutants in the central core residues of LolE and LolC rescued by arabinose-induced protein expression**. c,d** Western blot detection of wild-typeLolCDE and mutants in periplasmic residues of LolE (c) and LolC (d) as shown in (b**)**. **e** The interactions between the elbow helix in the N- terminus and C- terminus of LolE and the signature loop of LolD.

**Table S1 Cryo-EM data collection, refinement and validation statistics**

|  | Lipoprotein bound LolCDE in nanodiscs  (EMD-51520, PDB 9GRC ) | ATP bound LolCD^E171Q^E in nanodiscs  (EMD-51637, PDB 9GVK) |
| --- | --- | --- |
| **Data collection and processing** |  |  |
| Magnification | 130000 | 29000 |
| Voltage (kV) | 300 | 300 |
| Electron exposure (e^–^/Å^2^) | 56 | 64 |
| Defocus range (μm) | -1.0 to -2.8 | -1.0 to -2.8 |
| Pixel size (Å) | 0.93 | 1.014 |
| Symmetry imposed | *C1* | *C1* |
| Initial particle images (no.) | 1,398,358 | 1,281,615 |
| Final particle images (no.) | 192,234 | 268,413 |
| Map resolution (Å) | 3.55 | 3.25 |
| FSC threshold | 0.143 | 0.143 |
| Map resolution range (Å) | 3.2-5.6 | 3.0-7.5 |
| **Refinement** |  |  |
| Initial model used | 7ARH | 7ARK |
| Map sharpening *B* factor (Å^2^) | -108 | -60 |
| Model composition |  |  |
| Non-hydrogen atoms | 9717 | 9673 |
| Protein residues | 1263 | 1256 |
| Ligands | 1 | 1 |
| *B* factors (Å^2^) |  |  |
| Protein | 33.21 | 42.33 |
| Ligand | 34.87 | 103.93 |
| R.m.s. deviations |  |  |
| Bond lengths (Å) | 0.002 | 0.004 |
| Bond angles (°) | 0.597 | 0.713 |
| **Validation** |  |  |
| MolProbity score | 1.67 | 1.83 |
| Clashscore | 7.37 | 8.11 |
| Poor rotamers (%) | 0.17 | 0.39 |
| Ramachandran plot |  |  |
| Favored (%) | 96.08 | 94.30 |
| Allowed (%) | 3.92 | 5.70 |
| Disallowed (%) | 0.00 | 0.00 |
